# Predicting the demand for preschool teachers in China under the three-child policy: a comparative analysis between Guangdong Province and Jilin Province

**DOI:** 10.3389/fpsyg.2025.1536476

**Published:** 2025-05-09

**Authors:** Dongyuan Xu, Zhichao Wang, Jiwei Zhang

**Affiliations:** ^1^Faculty of Education, Northeast Normal University, Changchun, Jilin, China; ^2^Faculty of Education, Key Laboratory of Applied Statistics of MOE, Northeast Normal University, Changchun, Jilin, China

**Keywords:** three-child policy, preschool education, teacher demand, population prediction, LSTM neural network models

## Abstract

**Introduction:**

This study predicts the development trends of the scale of preschool children in kindergartens and the demand for full-time teachers in Guangdong Province and Jilin Province under the context of three-child policy, while exploring inter-provincial differences and their underlying reasons.

**Methods:**

Based on the statistical data released by the national and provincial regions, we constructed and trained two Long Short-Term Memory (LSTM) neural network models for prediction.

**Results:**

From 2023-2035, the scale of preschool children in kindergartens and the demand for full-time teachers in Guangdong Province exhibit an overall upward trend, while in Jilin Province, both exhibit an overall downward trend. The opposite development directions of the two provinces are the result of the combined effects of factors such as the level of economic development, population structure, and culture.

**Discussion:**

Our findings provide data support for local governments to formulate education policies that are coordinated with the changes in the school-age population and the demand for teachers. It also has reference significance for foreign countries.

## Introduction

In the context of ongoing adjustments to fertility policies, preschool education, as the forefront of the national education system, must promptly adjust the allocation of teacher resources according to dynamic changes in the population to meet the educational needs of school-age children in different regions. To address the “low fertility rate trap,” China has successively implemented policies such as two-child policy for couples both of whom are only children, two-child policy for one member of a couple, universal two-child policy, and three-child policy since 2011 (Sun and Wang, 2022). The gradual relaxation of birth restrictions has positively expanded citizens’ reproductive space and autonomy (Ma, 2021). Changes in population size brought about by fertility policies will directly affect the number of school-age children in preschool education and the demand for teacher resources. Especially in different provinces, due to differences in economic development levels, population bases, cultural customs, and other aspects, the degree of population growth stimulated by fertility policies varies among provinces, thereby affecting the demand for teacher resources for school-age children in preschool education. However, by searching databases, we found that most studies focus on predicting the scale of preschool children and the demand for educational resources at the national level or in a specific region (Zhang et al., 2013; Shi, 2017; Huang et al., 2023), with few studies conducting horizontal comparative analysis at the provincial level. Considering the high degree of coupling between population and education, a comparative study of Guangdong Province and Jilin Province, as typical representatives of China’s high fertility potential area and low fertility inertia area, can better reveal the impact of different population development models stimulated by fertility policies on the allocation of educational resources. Therefore, this study selects the above two “extreme” provinces as research samples. By constructing Long Short-Term Memory (LSTM) neural network models, we predict and analyze the trends and differences in the scale of preschool children and the demand for full-time teachers in Guangdong Province and Jilin Province from 2023 to 2035 under the context of the three-child policy. The aim is to provide data support for relevant departments to plan preschool education development and adjust the allocation of teacher resources, which is also of reference significance for foreign countries.

### The impact of population changes on the demand for teacher resources

“Almost all aspects of educational development planning are closely related to population size, structure, and distribution, and dynamic changes in population must be fully considered” (Ta Ngoc Châu, 1969). In the 1960s, the rise of women’s liberation movements in the United States led to a decline in fertility rates. By 1974, the number of births had decreased by 27% (Glenny, 1980), significantly reducing the scale of preschool education and thus lowering the demand for preschool teachers. Since the 1970s, Japan has entered a society characterized by low fertility rates, directly leading to a year-by-year decrease in the number of preschool children (Wang and Liu, 2020) and significantly impacting the allocation of resources such as teachers and funding. To address the phenomenon of “low fertility,” China implemented the universal two-child policy in 2016. The positive effects of this policy significantly increased the number of preschool enrollments and the demand for teachers in 2019. However, the policy’s effect gradually weakened in the following years. To once again stimulate the social fertility desire, China introduced the three-child policy in 2021. The effect of the new fertility policy on population growth has regional characteristics (Chen and Zhou, 2024). Guangdong Province, the most populous province in China, had a birth rate of 8.30‰ in 2022, with 4,9,805 million preschool children and a supply of 340,200 full-time teachers (Department of Development and Planning of Chinese Ministry of Education, 2023). In contrast, in the severely depopulated northeastern region, Jilin Province has the smallest population, with a birth rate of 3.34‰ in 2022, 400,100 preschool children, and a supply of 31,300 full-time teachers (Department of Development and Planning of Chinese Ministry of Education, 2023). Different population change trends and growth rates will inevitably have varying impacts on the population size at the preschool education stage, thereby affecting the supply pattern and spatial distribution of teacher resources and bringing new challenges to the high-quality development of provincial preschool education.

### Research on the scale of preschool children and the demand for teacher resources based on population predictions

Most Chinese scholars predict the scale of preschool children and the demand for teachers based on the context of fertility policies and using different geographic ranges as samples. At the national level, Yang et al. (2016) using the universal two-child policy as the context, predicted the scale of preschool children from 2016 to 2035 using China Population Prediction Software (CPPS software). The results exhibited that the preschool education scale presented an “A-shaped” change, with 2021 as the dividing point, and the demand for educational resources such as teachers and kindergartens increased accordingly. Li and Shi (2024) considering the impact of the three-child policy on fertility rates, used CPPS software to predict that the number of births in China from 2021 to 2035 would exhibit a “U-shaped” change, with the number of preschool children and teacher demand both exhibiting a downward trend. At the provincial or municipal level, Liu and Han (2019) used the Leslie matrix model to predict the scale of urban and rural preschool education and the demand for teachers in Gansu Province from 2019 to 2035 under the universal two-child policy. The results exhibited that the number of preschool children and the demand for teachers both exhibited a trend of first increasing and then decreasing. Zeng (2021) using the Logistic model and Gray Model First-Order One Variable [GM (1,1) model], predicted the population size and preschool education resources in Chongqing under the universal two-child policy and found that there would be no contradiction between the supply and demand of kindergarten teachers in the coming years. Additionally, some scholars have conducted prediction studies for other regions (Shi, 2020; Wang, 2023).

Foreign scholars have earlier explored the impact of population changes on the allocation of educational resources. Thomas (1984) studied the relationship between population size and teachers, finding that the scale of pupils and the number of teachers changed in the same direction. When fertility rates decline, the scale of pupils shrinks, and the demand for teachers decreases, which may cause an imbalance in the age structure of teachers. Briault (1986) indicated that a decline in fertility rates would reduce the demand for educational resources to a certain extent, affecting the allocation of educational resources. When fertility levels drop, the demand for education is less than the supply, which may lead to an oversupply of educational resources and the phenomenon of education marketization; when the demand for education exceeds the supply, it may cause a shortage of educational resources, leading to competition and panic.

Overall, academic research on population changes and the demand for educational resources has various focuses and different prediction methods. While existing research provides important academic references for this study, there are also some deficiencies. Firstly, regarding the research sample, most domestic scholars choose the country or a specific province or city as the scope to predict the scale of preschool children and the demand for educational resources under fertility policies, with few scholars conducting horizontal comparative studies at the provincial level. Secondly, in terms of the research context, due to the earlier publication time, most scholars have conducted predictive analysis based on the universal two-child policy, without considering the new impact of the recently released three-child policy on the future scale of preschool children and the demand for teacher resources, thus lacking timeliness and credibility. Thirdly, regarding the prediction methods, CPPS software requires setting fixed parameters, with limited flexibility and adaptability in population prediction; the Logistic model is not ideal for long-term time series data prediction (Booth, 2006); the GM(1,1) model relies on the quality of historical data, and its prediction accuracy decreases with the increase of data dispersion (Chen et al., 2021). LSTM neural networks (Hochreiter and Schmidhuber, 1997) have often proven to be more power (Piech et al., 2015), and are highly advantageous in mining long-term dependencies in time series data (Yang and Wang, 2019). They have been applied to prediction studies on students’ academic performance (Kukkar et al., 2024), teachers’ career development (Fan, 2024), and population (Riiman et al., 2019).

Therefore, in order to make up for the shortcomings of the above studies, this study selects Guangdong Province and Jilin Province, which have significant differences in economic development levels and population sizes, as research samples. Using the more robust and flexible LSTM neural network models to predict and analyze the trends and differences in the scale of preschool children and the demand for full-time teachers in these two provinces from 2023 to 2035 under the context of the three-child policy.

### The context of this study

Since the 21st century, the high-quality development of preschool education has been regarded by the international community as an important indicator of national wealth (Huang and Xiong, 2021). Changes in the scale of the birth population brought about by fertility policies may exacerbate the supply–demand imbalance of teacher resources, leading to shortages or wastage of teacher resources, which in turn affects the high-quality development of preschool education. At the National Education Work Conference held in 2024, China emphasized the need to “focus on population change trends, strengthen the forward-looking layout of basic education” and “optimize the allocation of teacher resources to support the strength of education with strong teachers” (Ministry of Education, 2024). In preschool education resources, a reasonable and adequate supply of teachers is the prerequisite for ensuring that every child enjoys the right to equal education and is also a key condition for smoothly carrying out daily educational activities. The children-to-teacher ratio is an important indicator of the level of teacher resources. According to the “Educational Statistics Yearbook of China,” the average children-to-teacher ratio in preschool education in Guangdong Province over the past three years was 15:1, and in Jilin Province, it was 13:1, which is significantly lower than the children-to-teacher ratios of 10:1 or 9:1 in developed countries (Wang et al., 2023). Additionally, there are disparities in teacher resources between provinces. Therefore, under the context of the three-child policy, reasonably allocating provincial teacher resources based on dynamic population changes has become an important strategy to promote balanced and high-quality development of preschool education.

The following research questions will guide the prediction and analysis of the scale of preschool children and the demand for full-time teachers in Guangdong Province and Jilin Province:

1. How will the scale of preschool children in Guangdong Province and Jilin Province change from 2023 to 2035, and what development trends will it exhibit?
2. How will the demand for full-time teachers in preschool education in Guangdong Province and Jilin Province change from 2023 to 2035, and what development trends will it exhibit?
3. Are there significant differences in the changing trends of the scale of preschool children and the demand for full-time teachers between Guangdong Province and Jilin Province from 2023 to 2035? What are the reasons for these differences?

## Methods

### Data preparation and preprocessing

#### Data sources

The basic data on the current state of preschool education in Guangdong Province and Jilin Province were sourced from the “Educational Statistics Yearbook of China.” Birth population data were primarily compiled from the “Statistical Bulletin of National Economic and Social Development” published annually by each province. All data is publicly available statistics from official websites and publications, with consistent statistical standards and a continuous time span. Considering the availability and timeliness of the data and the timing of the promulgation of the fertility policy, the data for Guangdong Province were intercepted from 2012 to 2022; because of the fluctuation of the data for Jilin Province, the data were selected from 2010 to 2022 to capture the long-term trend more accurately.

#### Selection of feature variables

The quality, quantity, and correlation of feature variables directly affect the predictive ability of the models. According to the relevant provisions of the “Kindergarten Staffing Standards (Interim)” and supplemented by Pearson correlation test results, we ultimately selected the birth population, the number of children in kindergartens, the number of classes, and the children-to-teacher ratio as the variables influencing the demand for full-time preschool teachers.

#### Data cleaning and preprocessing

Data cleaning and preprocessing are critical steps in the machine learning process. These steps improve data quality and adapt the data to the models’ requirements through various operations such as cleaning and transformation. The specific steps are as follows:

(1) Outlier detection using box plots.
(2) Applying min-max normalization to scale the raw data to a similar range.
(3) Establishing mapping relationships between input data and output data using sliding window.

### Model construction

**Figure 1:**
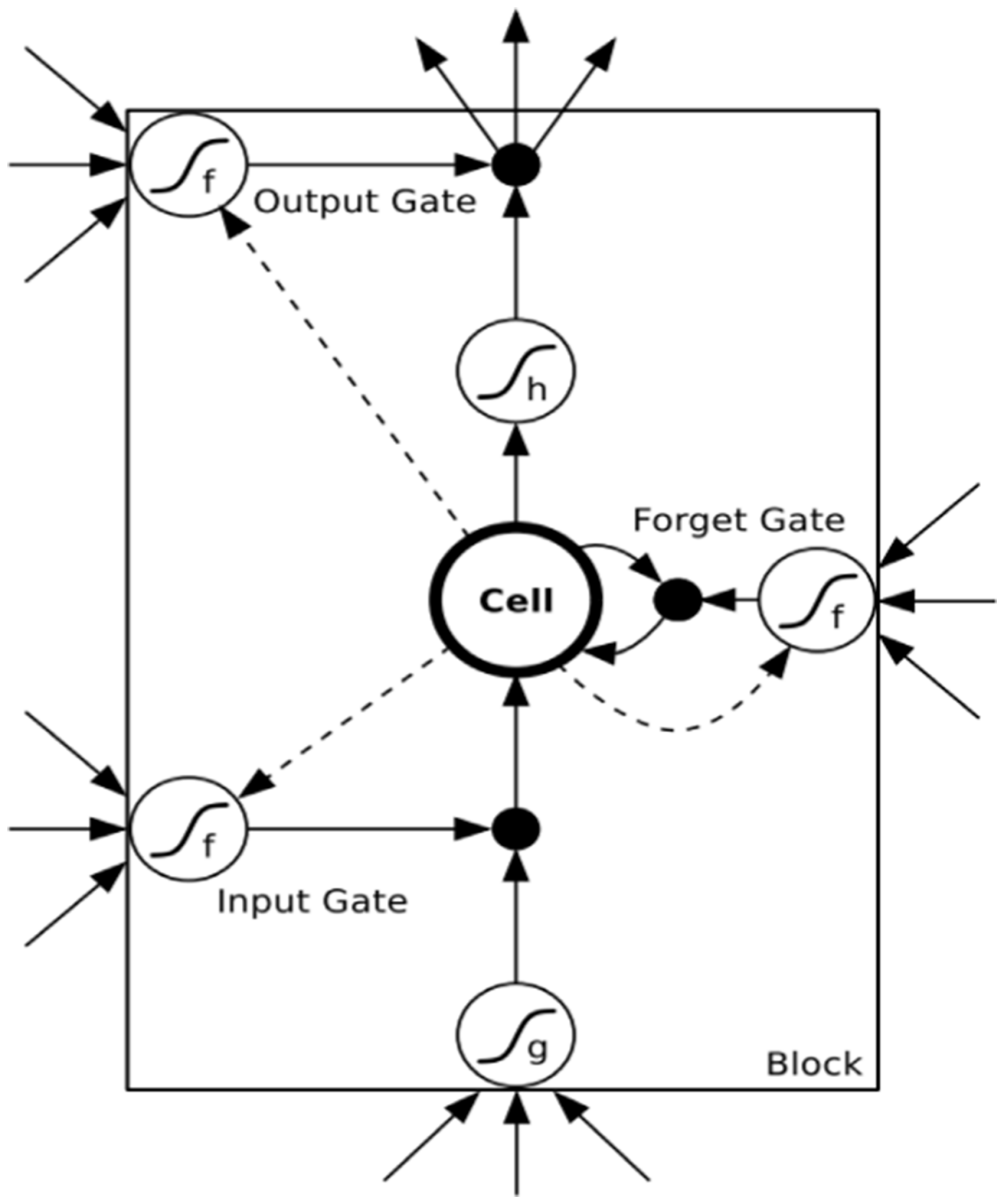
Neuron structure.

The LSTM neural networks are variants of the Recurrent Neural Network (RNN) that effectively address the problem of gradient vanishing caused by increased network layers and the passage of time (Zhou and He, 2023). The structure of each neuron in LSTM neural networks, as exhibited in Figure 1 (Graves, 2012), includes a cell state and three gating structures.

Based on the TensorFlow and Keras frameworks, this study constructed LSTM neural network models. The hyperparameters of the models are set as follows:

(1) Two-layer LSTM neural networks are constructed, with the number of neurons in each layer being 32 and 64, respectively.
(2) A fully connected layer with 5 neurons is added to the output layer.
(3) The optimizer chosen is the Adaptive Moment Estimation (Adam) algorithm, which can adaptively design learning rates for different parameters.
(4) The loss function is Mean Squared Error (MSE).
(5) An early stopping strategy is adopted to address the overfitting problem that may occur during optimization. This is achieved by setting “monitor,” “min_delta,” and “patience” to stop the training early, with specific parameter settings of {MSE, 0.001, 15}.
(6) According to the goodness of fit, the optimal time window for the models is determined to be 2*5, meaning that the data from the previous 2 years are used to predict the data for the next year.

### Model training

Before training the models, the dataset needs to be divided into a training set and a test set in chronological order. The training set is used to train parameters so that the models can capture the features and distribution patterns of the data. The test set is used to evaluate the models’ generalization ability on unseen data.

During the training process, GridSearchCV is used to exhaustively traverse all hyperparameter combinations to find the optimal configuration for “learn_rate,” “batch_size,” and “epochs.” Additionally, the datasets need to be input into the models multiple times, and iterations are stopped once the loss function converges and stabilizes to prevent underfitting due to too few iterations.

### Model validation

#### Model fit tests

**Figure 2:**
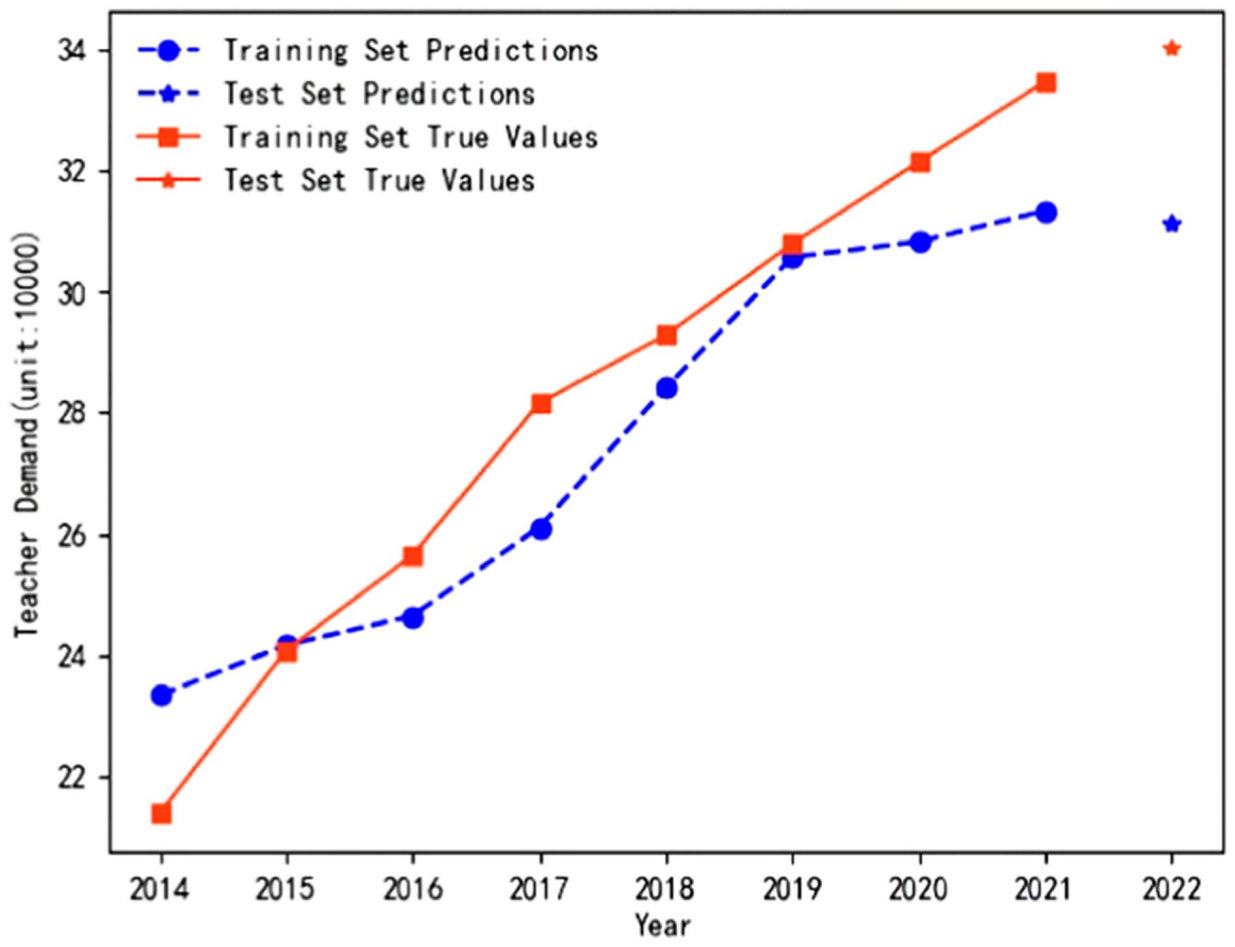
Fitting effect of the model in Guangdong Province.

**Figure 3:**
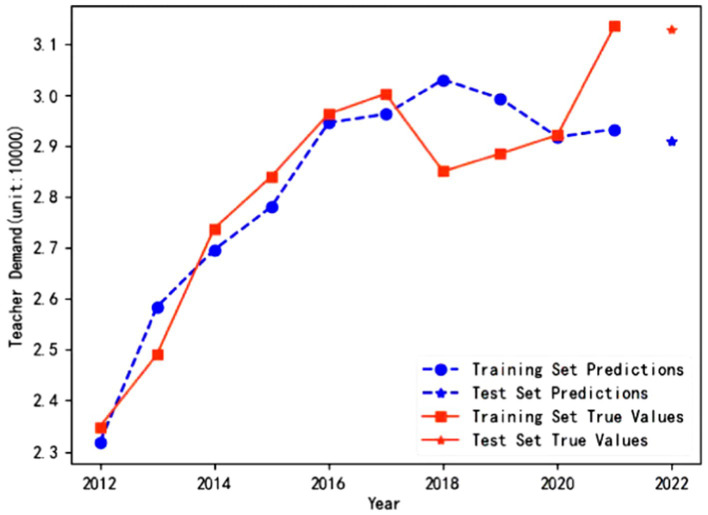
Fitting effect of the model in Jilin Province.

The trained and optimized LSTM neural network models are used to predict the demand for full-time preschool teachers in Guangdong Province and Jilin Province to test the fitting effects. As shown in Figure 2, the prediction curves of the model on the training and test sets closely match the actual trends in the demand for full-time teachers, with no significant deviations observed. Figure 3 shows that the predicted values fit well with the true values from 2012 to 2017. Although slight deviations occurred in 2018 and 2019, a similar trend is restored after 2020. Therefore, it can be shown that LSTM neural network models have good stability in long-term predictions.

#### Model evaluation metrics

According to Hyndman and Koehler (2006), we chose scale-dependence metrics to assess the absolute value of the models’ errors, including Mean Absolute Error (MAE), Mean Square Error (MSE), and Root Mean Square Error (RMSE). In addition, we report *R*-squared (*R*^2^) to validate the explanatory power to provide a comprehensive measure of the models’ predictive accuracy. The values of the evaluation metrics are exhibited in Table 1.

**Table 1:** Evaluation results of the models for Guangdong Province and Jilin Province.

Province	MAE	MSE	RMSE	*R* ^2^
Guangdong	0.0839	0.0099	0.0994	0.8391
Jilin	0.0632	0.0066	0.0815	0.7466

Mean Absolute Error, MSE, and RMSE are used to measure the deviation between the predicted values and the actual values; the smaller the values, the higher the prediction accuracy. *R*^2^ is used to measure the extent to which the models explain the variability of the dependent variable; the closer the value is to 1, the better the models’ fitting effect. As exhibited in the table above, the MAE, MSE, and RMSE values are all below 0.1, and the *R*^2^ values are all above 0.7. This indicates that the models have small prediction errors and a high degree of fit.

## Results

### Prediction of preschool children scale in Guangdong Province and Jilin Province

The “Kindergarten Work Regulations” consider children aged 3–6 years as the appropriate population for preschool education (Ministry of Education, 2016). Therefore, this study predicts the number of 3–6-year-old children actually enrolled in kindergartens.

**Figure 4:**
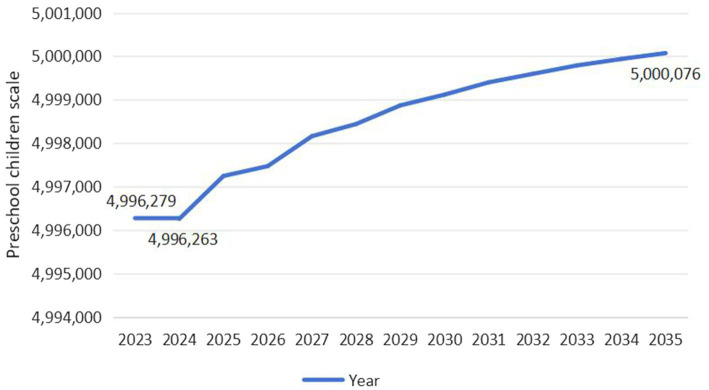
Predicted trend of preschool children scale in Guangdong Province (Unit: Person).

**Figure 5:**
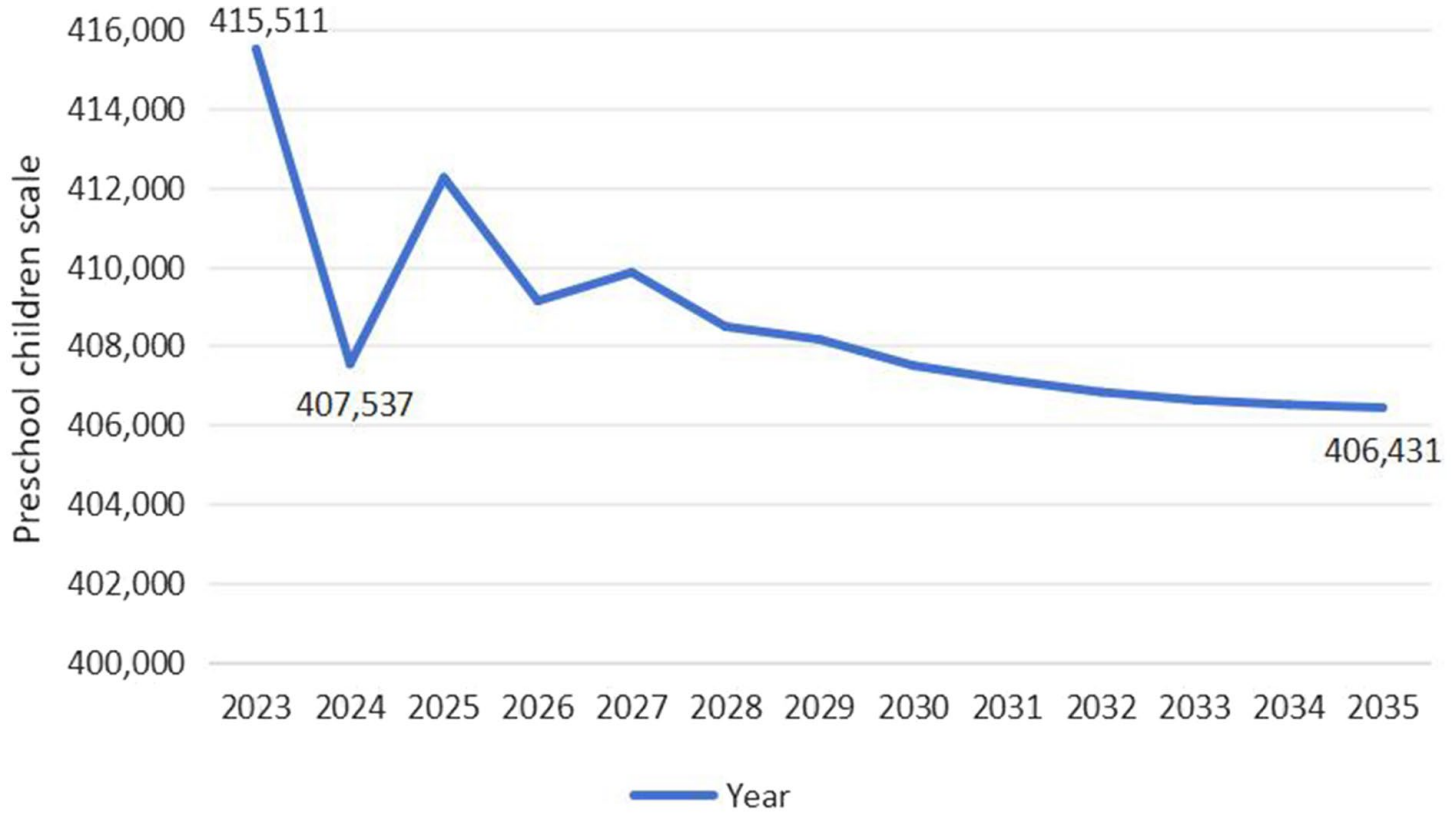
Predicted trend of preschool children scale in Jilin Province (Unit: Person).

As exhibited in Figure 4, the scale of preschool children in Guangdong Province exhibits an overall upward trend, expected to increase from 4,996,279 in 2023 to 5,000,076 in 2035, with a growth rate of 0.08%. Specifically, the scale is expected to drop to its lowest level in 2024, at 4,996,263, and then fluctuate upward, reaching its highest level by 2035. As exhibited in Figure 5, the scale of preschool children in Jilin Province exhibits a wave-like development trend, initially declining rapidly, then briefly recovering, and finally decreasing year by year. It is projected to decrease from 415,511 in 2023 to 406,431 in 2035, with a decline rate of 2.19%. Specifically, the scale is expected to decrease from 415,511 to 407,537 between 2023 and 2024, a reduction of 1.92%. However, after 2024, the scale will temporarily recover to 409,855 by 2027, followed by a continuous decline, reaching its lowest point in 2035.

### Prediction of the demand for full-time preschool teachers in Guangdong Province and Jilin Province

**Figure 6:**
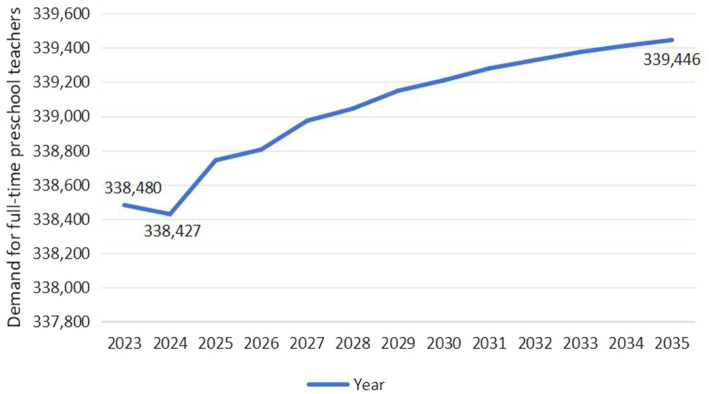
Predicted trend of demand for full-time preschool teachers in Guangdong Province (Unit: Person).

**Figure 7:**
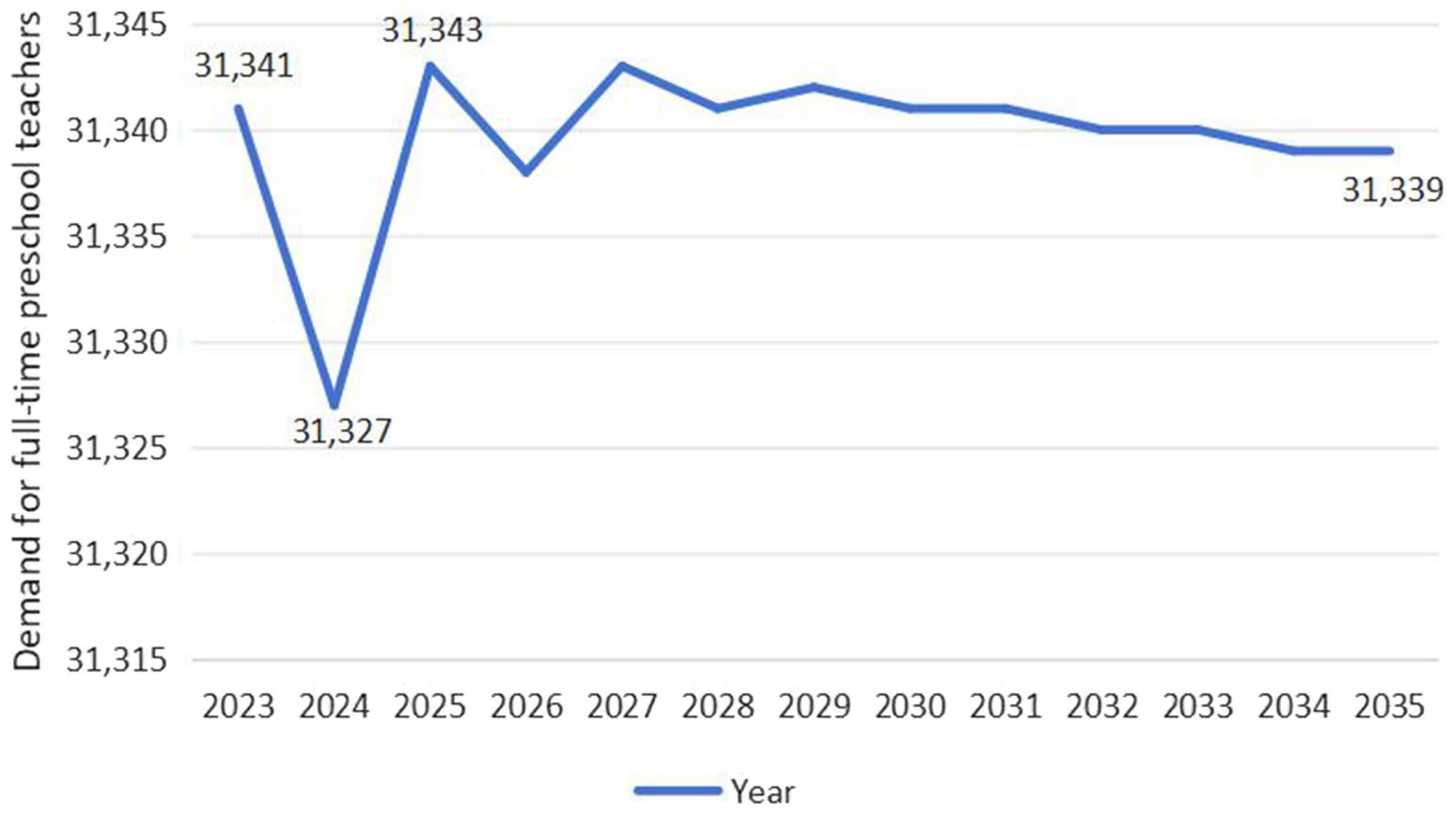
Predicted trend of demand for full-time preschool teachers in Jilin Province (Unit: Person).

As exhibited in Figure 6, the demand for full-time preschool teachers in Guangdong Province exhibits an overall upward trend, projected to increase from 338,480 in 2023 to 339,446 in 2035, with a growth rate of 0.29%. Specifically, the demand for full-time teachers is expected to drop to its lowest level in 2024, at 338,427, and then fluctuate upward, reaching its highest level by 2035. As exhibited in Figure 7, the demand for full-time preschool teachers in Jilin Province exhibits a wave-like development trend, initially declining, then rising, and finally slowly falling. It is projected to decrease from 31,341 in 2023 to 31,339 in 2035. Specifically, the demand for full-time teachers is expected to decrease from 31,341 to 31,327 between 2023 and 2024. However, after 2024, the demand will temporarily rise to 31,343 by 2027, followed by a year-by-year decline.

## Discussion

This study constructed LSTM neural network models to predict and analyze the trends and differences in the scale of preschool children and the demand for full-time teachers in Guangdong Province and Jilin Province from 2023 to 2035, under the context of the three-child policy. In this section, we discuss the research results, revealing the development process of preschool children scale and full-time teacher demand in these two provinces over the next 13 years, and explore the differences and causes of these differences.

### The overall scale of preschool children in Guangdong Province and Jilin Province exhibits opposite trends

China’s current population development is characterized by low fertility, aging, and regional population differentiation (Chen and Zhou, 2024). This indicates that, against the backdrop of continuously declining fertility rates and deepening aging, the development trends and change rates of populations in different regions vary. This conclusion supports our research results: the scale of preschool children in Guangdong Province, located in the eastern region, exhibits an overall growth trend, while Jilin Province, located in the northeast region, exhibits the opposite trend. Specifically, between 2023 and 2024, the decline in the birth population due to the fading effect of the universal two-child policy caused a reduction in the preschool children scale in both provinces to varying degrees. However, after 2024, Guangdong Province and Jilin Province exhibit opposite development trends, indicating that the effect of the three-child policy varies between regions. The extent to which fertility policies stimulate population growth is related to the level of economic and social development, as well as the combined effects of population structure, culture, and lifestyle (Chen, 2021). The continuous emergence of new industries attracts a large number of young people to migrate to Guangdong. According to statistics, Guangdong Province has recorded an average annual net migration increase of 56,900 persons since 2016. This pattern of population development has continued to raise the total population while expanding the original childbearing age group. Additionally, some areas in Guangdong still retain the traditional belief that “more children bring more blessings,” providing some flexibility for the fertility policy. Yang (2024) points out that the future population development in Guangdong Province is characterized by a large total population that will continue to grow for some time. Wu and Yu (2024) believe that in the face of the reality of the overall low social fertility desire, the three-child policy will not have much room to stimulate population growth. The number of births will have a delayed impact on the scale of preschool children. Therefore, the prediction that the scale of preschool children in Guangdong Province will exhibit a low-speed growth trend in the future is consistent with existing research results. Jilin Province, as one of China’s traditional heavy industrial bases, has long relied on traditional industries for its economic development. The difference in the economic structure is the most crucial factor leading to population loss (Zhao and Liu, 2018). In addition, the northeast region is deeply affected by the dual impacts of long-term negative natural population growth and net decline in the migrant population (Yuan and Wang, 2024). Since the promulgation of the universal two-child policy, the number of births in Jilin Province has been decreasing by an average of 15,100 persons per year, a rate of decline that is among the higher levels in China. In this context, the positive effects of the three-child policy are unlikely to change the “low fertility” phenomenon in the short term. As the least populous province in the northeast, the trend of the preschool children scale in Jilin Province, exhibiting an initial rise followed by a decline after 2024, is also consistent with existing research results.

Differences in the degree of response to fertility policies directly affect the number of births and the scale of the preschool-age population in different regions. Therefore, local governments should establish scientific population forecasting systems, dynamically monitor the scale of the preschool-age population, and adaptively allocate teacher resources to meet the actual educational needs of the population based on the implementation effects of the three-child policy and the distribution of current educational resources. Additionally, for Jilin Province, which is facing the pressure of double population reduction, in order to achieve the expected effects of the three-child policy, government should not only actively implement the policy but also further improve the fertility environment, build a new culture of marriage and childbirth (Feng, 2021). Through various forms of active publicity, a positive and healthy fertility culture should be gradually created to guide the people of childbearing age in increasing their fertility desire.

### Guangdong Province and Jilin Province should further expand full-time teacher resources to promote high-quality development of preschool education

The children-to-teacher ratio is one of the important indicators for measuring the adequacy of teacher resources. In Oklahoma, USA, the preschool education children-to-teacher ratio is stipulated to not exceed 10:1, and in Finland, the ratio for children aged 3–5 is set at 7:1 (Li and Shi, 2024). In China, the “Interim Standards for Kindergarten Staffing” specifies an average children-to-teacher ratio of 15:1 in kindergartens, with a high standard ratio of 10:1. According to the prediction results, by 2035, the children-to-teacher ratio in Guangdong Province will be 14.68:1, and in Jilin Province, it will be 12.71:1. These data indicate that while both provinces meet the basic standard, they fall short of the high standard and are at a lower level compared to developed countries. This suggests that in actual educational practice, both Guangdong and Jilin Provinces experience “overcrowding,” where the number of full-time teachers cannot meet the educational needs of the children in kindergartens, resulting in a certain level of supply pressure on teacher resources in the future.

Under the goal of building a strong educational nation, local governments should actively eliminate the phenomena of “high children-to-teacher ratios” and “large class sizes” in their pursuit of high-quality development of preschool education. This can be achieved through joint efforts in policy, finance, and human resources to effectively supplement the preschool teacher workforce. Additionally, high-quality teachers are crucial for improving preschool education quality. The quality and teaching skills of teachers directly determine the level and quality of education that children receive. Therefore, it is essential to strictly implement the standards for hiring preschool teachers, clearly specifying the requirements for new teachers in terms of subject knowledge, professional skills, and educational qualifications. Moreover, during pre-service training and in-service training, emphasis should be placed on enhancing professional knowledge and skills, as well as improving educational concepts and practical abilities. This ensures that every teacher is capable of fulfilling their educational duties, providing each child with high-quality teachers and educational services.

### The overall demand for full-time preschool teachers in Guangdong Province and Jilin Province exhibits opposite trends

The differentiation in regional population increases and decreases will significantly impact the scale and structure of the school-age population, directly influencing the supply patterns and spatial distribution of teacher resources (Wu et al., 2023). According to the prediction results, the demand for full-time preschool teachers in Guangdong and Jilin Provinces follows the same trend as the changes in the scale of preschool children, consistent with Thomas’s (1984) conclusions about the relationship between population and teachers. Overall, the demand for full-time preschool teachers in Guangdong Province exhibits an upward trend, while Jilin Province exhibits a downward trend. Specifically, between 2023 and 2024, the reduction in the scale of preschool children led to a corresponding decline in the demand for full-time teachers in both provinces. However, after 2024, Guangdong Province exhibits an annual growth trend, whereas Jilin Province exhibits a pattern of fluctuating increases followed by a slow decline from 2024 to 2035. The scale and structure of the school-age population are crucial for the allocation of teacher resources. Regardless of the educational stage, the scale of students is the key factor determining the demand for teachers. Therefore, the differences in the development of demand for full-time preschool teachers between the two provinces are largely due to the varying scales of preschool children. It is noteworthy that from 2023 to 2035, the growth rate of demand for full-time preschool teachers in Guangdong Province exceeds the growth rate of preschool children, while the decline rate of demand for full-time preschool teachers in Jilin Province is less than the decline rate of preschool children. This is because the “China Education Modernization 2035” plan proposes to provide quality preschool education by 2035, with sufficient and reasonable teacher allocation and better children-to-teacher ratios being fundamental to the high-quality development of preschool education. Therefore, even in the face of population reduction, the demand for full-time teachers will not decrease significantly.

Local governments need to accurately understand the impact of the three-child policy on the demand for preschool education in their regions and plan the allocation of teacher resources in advance. For regions where the current stock of teachers cannot meet future preschool education demands, the government should diversify teacher supplementation channels, such as encouraging normal students to undertake teaching internships, incentivizing surplus primary and secondary school teachers to exchange and rotate, and implementing public-funded normal student orientation training programs to expand teacher resources (Huang and Li, 2023). For regions where teacher resources can meet future preschool education demands, the government should increase support for the continuing education and professional development of preschool teachers to improve the overall quality of the teaching staff. Additionally, the government should enhance the flexibility of teacher resource allocation to ensure quick adjustments to changes in future educational demands, thereby guaranteeing educational quality and equity.

## Conclusions, limitations, and implications

Under the context of the three-child policy and based on national statistical data, this study constructed LSTM neural network models to predict and analyze the development trends and differences in the scale of preschool children and the demand for full-time teachers in Guangdong Province and Jilin Province from 2023 to 2035. The results exhibit that the scale of preschool children and the demand for full-time teachers in Guangdong Province exhibit an overall upward trend, while Jilin Province exhibits the opposite trend. Additionally, we found that both provinces will need to further expand their full-time teacher resources to alleviate supply pressure in the pursuit of high-quality development with better children-to-teacher ratios in the future.

However, our study has some limitations. Firstly, due to space constraints, we selected Guangdong and Jilin provinces as the subjects of our study. Although this study has revealed some of the differences between the two provinces in terms of the scale of preschool children and the demand for full-time teachers, given the complexity and diversity of population changes, the sample size should be expanded in future studies to improve the generalizability and representativeness of the research. Secondly, population development is the result of economic, social, cultural, and other factors combined. It is difficult to include all influencing factors in the models in a relatively stable manner, which may cause slight prediction errors. In the future, factors such as economic development indicators (e.g., GDP growth rate), population migration rates, and the number of women of childbearing age can be introduced into the models to compensate for the blind spots in the existing data. Additionally, a panel database can be established to periodically compare the actual values with the predicted values to verify the practical application of the models. Lastly, the non-linear characteristics of population development often cause the actual population change to exceed or lag behind the predicted results, which is also an inevitable problem in the process of population prediction. Since the expected effect of the three-child policy has not yet been realized, in order to eliminate the impact of the lagging effects of the policy on the prediction results, it is necessary to incorporate the latest data into the models in the future to comprehensively capture the underlying trends in population development.

Despite these limitations, our study still holds theoretical and practical significance. In terms of theoretical value, through a literature review, we found that most existing studies choose to predict the scale of preschool children and the demand for teacher resources under fertility policies or urbanization for the entire country or specific provinces/cities. Few studies conduct horizontal comparative analysis between provinces. Therefore, our prediction study of preschool education in Guangdong Province and Jilin Province can enrich existing research perspectives and provide relevant research ideas. Additionally, we selected the LSTM neural networks as the prediction models, which can, to some extent, overcome the impact of non-linear population changes on prediction results, providing flexibility and robustness. In terms of practical value, “low fertility” is a challenge faced by many countries today. To mitigate the social problems caused by low fertility rates, many countries have introduced fertility policies encouraging childbirth. The implementation of such policies will inevitably lead to population changes, affecting the demand for teacher resources at various educational stages. The uneven distribution of preschool teacher resources is a phenomenon not only in China but also abroad. Therefore, selecting Guangdong Province and Jilin Province, which have significant differences in economic development levels, culture, and population size, as research samples to analyze the impact of population changes on the demand for preschool teacher resources is more representative. This will help local governments plan the allocation of preschool teacher resources proactively, promoting high-quality development of preschool education.

## Data Availability

Educational data used in this study was obtained from publicly available books and can also be accessed from the following link: http://www.moe.gov.cn/jyb_sjzl/moe_560/2022/. Population data is sourced from the official websites of the provincial governments, which can be accessed from the following links: http://www.gd.gov.cn/ and http://www.jl.gov.cn/.
